# The integrated care costs of HIV and non-communicable diseases in South Africa

**DOI:** 10.5588/pha.24.0027

**Published:** 2024-12-01

**Authors:** M. Moyo-Chilufya, T. Mgutshini, A. Musekiwa, C. Hongoro

**Affiliations:** ^1^ School of Health Systems and Public Health, Faculty of Health Sciences, University of Pretoria, Pretoria, South Africa;; ^2^ University of South Africa, College of Graduate Studies, Pretoria, South Africa;; ^3^ Human Sciences Research Council, Pretoria, South Africa.

**Keywords:** HIV, non-communicable diseases, integrated, Southern Africa, cost

## Abstract

**SETTING:**

In sub-Saharan Africa, the syndemic of HIV and non-communicable diseases (NCDs) poses a significant challenge. To address this, leading global think tanks like the WHO advocate for integrated HIV/NCD care at primary healthcare levels. However, comparative empirical data on the costs of integrated care are limited. South Africa, with the largest HIV programme globally, was purposively selected for our comparative case study.

**OBJECTIVE:**

To determine the cost of integrated HIV/NCD care from the providers’ perspective at two ‘ideal status’ public healthcare facilities in South Africa as case studies.

**DESIGN:**

A multi-pronged methodology was used to collect provider cost data via retrospective documentary sources or records and a question-and-answer session with facility managers who provided key information on cost-related data. Data analysis utilised an activity-based costing (ABC) method.

**RESULTS:**

Despite the difference in the size of the clinics, the cost per patient in terms of ABC is similar between the two primary healthcare facilities, USD261.60 and USD226.30, respectively.

**CONCLUSION:**

The ABC method can be utilised to cost integrated care, foster health economic data availability for future research, and inform health policymakers.

## Abstract (fr)

**CONTEXTE:**

En Afrique subsaharienne, la synergie entre le VIH et les maladies non transmissibles (NCD, pour l’anglais « *non-communicable diseases* ») représente un enjeu majeur. Pour y faire face, des organisations de réflexion internationales, telles que l'OMS, recommandent l'intégration des soins pour le VIH et les NCD dans le cadre des soins de santé primaires. Néanmoins, les données empiriques comparatives concernant les coûts associés à ces soins intégrés demeurent insuffisantes. L'Afrique du Sud, qui abrite le plus vaste programme de lutte contre le VIH au monde, a été choisie intentionnellement pour notre étude de cas comparative.

**OBJECTIF:**

Évaluer le coût des soins intégrés VIH/NCD du point de vue des fournisseurs dans deux établissements de santé publique à statut optimal en Afrique du Sud.

**MÉTHODES:**

Une méthodologie à plusieurs volets a été utilisée pour collecter des données sur les coûts des prestataires par le biais de sources documentaires rétrospectives ainsi que sur des dossiers, complétée par une session de questions-réponses avec les responsables des établissements. Ces derniers ont fourni des éléments essentiels concernant les données de coûts. L'analyse des informations a été réalisée en utilisant une méthode de calcul des coûts basée sur les activités (ABC, pour l’anglais « *activity-based costing* »).

**RÉSULTATS:**

Bien que les cliniques varient en taille, le coût par patient en termes d'ABC est comparable entre les deux établissements de soins de santé primaires, s'élevant à 261,6 USD et 226,3 USD, respectivement.

**CONCLUSION:**

La méthode ABC peut servir à évaluer le coût des soins intégrés, à améliorer l'accès aux données économiques relatives à la santé pour des recherches ultérieures et à éclairer les décideurs dans le domaine de la santé.

Non-communicable diseases (NCDs) are, by proportion, the most lethal category of diseases.^1–4^ According to the WHO, NCDs were responsible for a staggering 17 million premature deaths among individuals under the age of 70 in 2021. Alarmingly, 86% of these premature deaths occurred in low and middle-income countries (LMICs), constituting a significant 71% of all global mortality.^1^

Notably, the comorbid presentation of NCDs with HIV/AIDS is a serious public health problem, as evidenced in a recent systematic review, which highlighted a substantial burden of NCDs among people living with HIV (PLHIV) in sub-Saharan Africa (SSA).^5^ The review reports noteworthy prevalence rates for depression (30.4%), hypertension (20.1%), chronic respiratory diseases (7.1%), diabetes (5.4%) and cervical cancer (1.5%) among PLHIV in SSA.^5^ The impact of the co-existence of HIV/AIDS with the NCDs in several countries in SSA, along with an acceptance of best-practice protocols, has provided the impetus for country health systems to integrate the management of major NCDs into HIV care. This approach leverages well-established HIV care platforms that present a unique opportunity to piggyback and incorporate NCD care services, thereby benefiting from the existing effectiveness of HIV care services. The benefits of this integrated approach are well specified in the literature and include enhanced operational efficiency, optimisation of synergistic effects, and the ability to address diseases that share common risk factors within a unified management framework.^2^ Despite these clear clinical benefits for SSA, little is known about the relative cost of integrated service models compared to parallel care options. To this end, the case for promoting integrated care platforms is incomplete. This observation is further supported by the WHO, which confirms a notable scarcity of data on the cost and cost-effectiveness of integrated NCD care within HIV care programmes, particularly in LMICs.^6,7^

South Africa is the country with the largest number of PLHIV, totalling 8.5 million. With an HIV prevalence rate of 14%, it ranks among the highest in SSA.^8^ Furthermore, South Africa records a significant proportion of NCD-related deaths, exceeding 50% in the general population.^9^ These disproportionately high rates of HIV and NCDs serve as a point of initial consideration for a clinical basis for integrated HIV and NCD care. Beyond the imperative of clinical outcomes, assessing the feasibility of integrated care models’ cost implications is important.

As an aid to this discourse, the WHO’s recently published comprehensive guidelines for the integration of NCD prevention and control within programmes related to HIV/AIDS, specifically highlights the key importance of financing as one of the five pivotal domains necessary for the successful integration of NCD services.^2^ Adequate budget preparations are strongly encouraged to ensure the availability of funding for the implementation of these integrated services. To facilitate these budget preparations, it is critical to clearly understand the costs associated with integrating NCD care into HIV care programmes.

Guided by these observations, the current study aims to bridge this knowledge gap by measuring the costs of providing integrated care services from the providers’ perspective. Additionally, it seeks to compare the costs between two ‘ideal clinics’^*^, an operationalised status of primary health care facilities that are only used in South Africa. By addressing these research objectives, this study sought to make a valuable contribution to the South African National Strategic Plan 2023–2028 that supports the integration for management of communicable and NCDs for PLHIV^10^ and sustainable development goal (SDG) 3.4, which strives to reduce premature mortality from NCDs by one third, through prevention and treatment while promoting mental health and well-being by the year 2030.^11^

## METHODS

### Study setting, design and participants

Due to the emphasis on integrated clinical services management at ideal clinics, we employed the purposive sampling method to select two primary health care facilities (ideal clinics) in Ekurhuleni municipality, serving as case studies for determining the cost of integrated HIV/NCD care in SSA from the provider’s perspective. Ekurhuleni is one of three metropolitan municipalities within the Gauteng province, with an approximate population of 3.2 million.^12^ The two ideal clinics are located in densely populated urban townships with a working age group (15–64 years) population of around 70%.

The clinics offer various services, including NCD management (for hypertension, congestive cardiac failure, chronic obstructive pulmonary disease, asthma, epilepsy, diabetes mellitus, and screening for cervical cancer), antenatal care, family planning, child health, acute services, and emergencies. They also provide mental health services and refer patients to secondary care when necessary. For laboratory services, these facilities rely on the National Health Laboratory Services (NHLS), which may offer mobile laboratory services or use a courier service to transport specimens to the central laboratory**.**

A multi-pronged approach was used to collect provider cost data: 1) secondary sources or records, and 2) health facility managers provided key information on cost-related data such as staff, patient, laboratory and drug costs. In cases where data was unavailable for one health facility, we used data from the other health facility, as both are publicly funded. Additionally, data were sourced from publicly available online resources and previous studies.

Integrated care services at the primary health care facilities were costing primarily via activity-based costing (ABC). Initially referenced as far back as 1949 by Goetz and subsequently refined by many, including Drury (2004)^13^ and Botha & Vermaak (2015),^14^ ABC is widely utilised globally and within the South African health system as a methodology to cost service delivery accurately. The approach is based on objectively allocating costs to key activities that relate to the care continuum of patients within specified care settings, e.g. 1) the cost of a bed, 2) the cost of human resource input in terms of medical and social care staff allocated to the care of a patient. The ABC approach helps managers understand the actual costs and cost drivers of activities within each health facility, facilitates informed decision-making, including cost allocation and cost reduction decisions, and improves budgeting and forecasting processes. Despite its numerous advantages, the ABC approach can be time-consuming and expensive to maintain and requires keeping a tab on the cost of each patient encounter within the health facility. Nonetheless, it is widely viewed as one of the most objective cost analysis methods for costing health care provision.

To determine the cost of integrated care at the primary health care facilities, we collected cost data on major components (personnel, drugs and other consumables and indirect/overhead costs), scale economies (size of programme/patients, number of staff, quantities of resources), scope economies (total cost of HIV and NCD care), efficiency (number of cases screened for the year), and proportion screened for NCDs. The data utilised were for 2022, and all pricing was calculated in 2022 United States Dollars (USD) (USD1 to South African rand 16.37). We ensured data quality by double entry onto Microsoft Excel spreadsheets, and the data was reconfirmed by health facility managers for accuracy before analysis.

To determine laboratory costs, we included the main tests utilised. For drug costs, only drugs that are utilised for first-line treatment without factoring in complications and combinations that may be prescribed depending on each patient’s needs were included.

We used cost accounting methods, apportionments to identify cost centres, and unit costs to analyse the data.^15,16^ We used a data extraction matrix tool for collecting supplementary data from the healthcare facilities and a checklist to assist with data collection for various categories.

### Ethical approval

Ethical approval for this study was granted by the University of Pretoria Faculty of Health Sciences Research Ethics Committee, Pretoria, South Africa (ref number: 591/2021), and permission was given by the Ekurhuleni Health District Research Committee, Ekurhuleni, South Africa (ref number: GP 202223_044). Health facility managers provided written consent to participate in the study and had the option to withdraw from the study at any point.

## RESULTS

### Personnel characteristics of the two ideal clinics

Table 1 shows the characteristics of the two clinics that provide integrated healthcare services as case studies. Clinic A boasts a larger staff contingent than Clinic B, predominantly comprising professional nurses, followed by administration clerks and data capturers. Both facilities host two sessional medical officers weekly. However, Clinic A provides a broader spectrum of on-site services compared to Clinic B. Notably, Clinic A accommodates weekly visits from a social worker, podiatrist, psychologist and psychiatrist, whereas Clinic B refers all patients requiring these specialised services to secondary facilities. There was no comparative data on staffing before integration.

**TABLE 1.** Personnel characteristics of the two ideal clinics

Variable	Clinic A	Clinic B
	*n* (%)	*n* (%)
Professional nurses	12 (22.6)	9 (30)
Administration clerks	8 (15.1)	5 (16.8)
Lay counsellors	5 (9.4)	4 (13.3)
Data capturers	5 (9.4)	3 (10)
General workers	5 (9.4)	—
Cleaners	4 (7.5)	4 (13.3)
Security officers	3 (5.7)	1 (3.3)
Enrolled nurses	2 (3.8)	2 (6.7)
Medical officers (sessional)	2 (3.8)	2 (6.7)
Junior pharmacy assistant	2 (3.8)	—
Social worker	1 (1.9)	—
Podiatrist	1 (1.9)	—
Psychologist	1 (1.9)	—
Psychiatrist	1 (1.9)	—
Pharmacy assistant	1 (1.9)	—
Total staff	53 (100)	30 (100)

### Patient consultations/screening

The Figure shows the number of patients per condition at the PHC facilities. Clinic A has a larger number of patients in care for HIV/AIDS compared to Clinic B. However, Clinic B has a higher number of patients with diabetes and hypertension. Notably, mental illness consultations are remarkably higher at Clinic A than at Clinic B. Furthermore, patients are routinely screened for HIV, hypertension, diabetes and chronic respiratory diseases.

**FIGURE.**
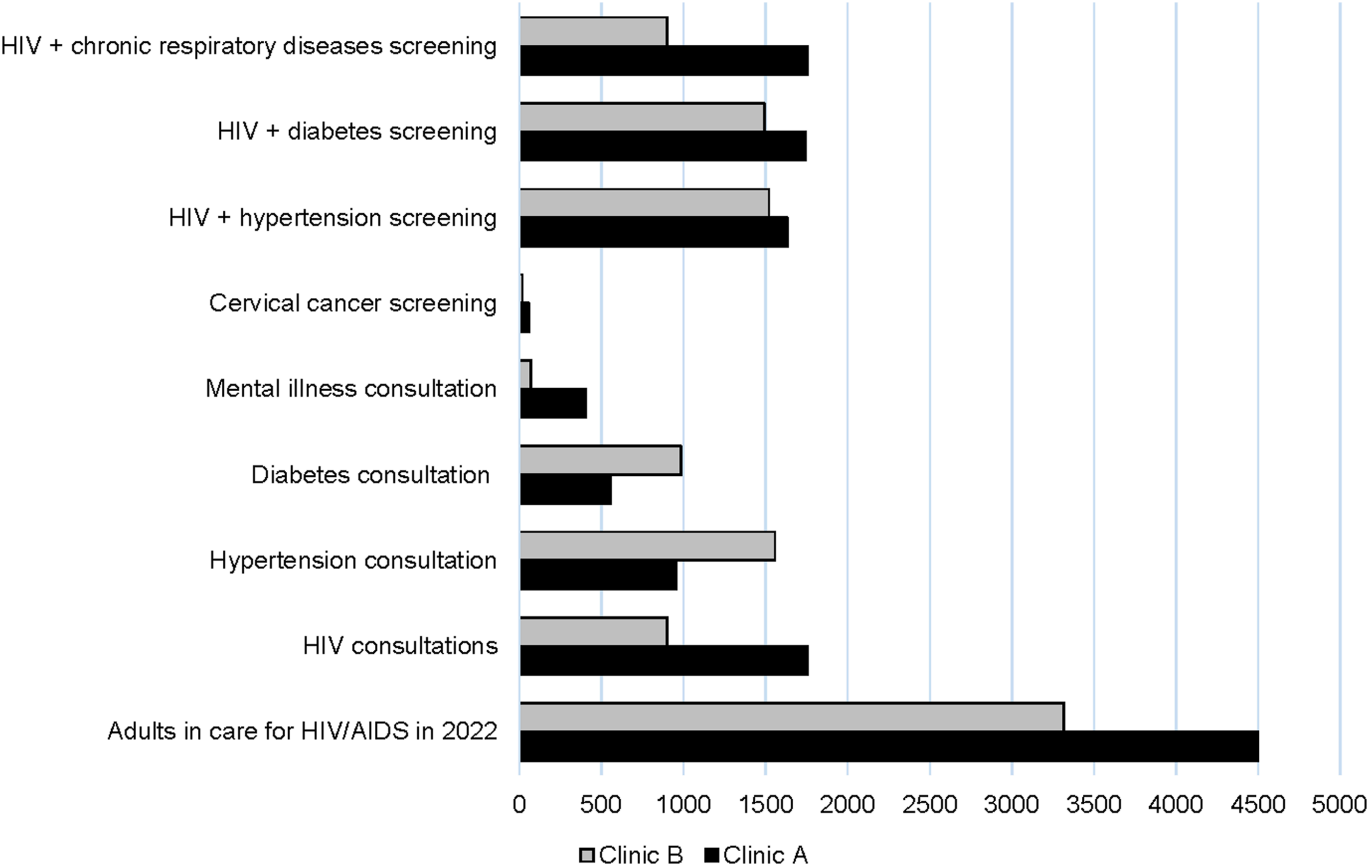
Patient consultations/screenings per month associated with integrated HIV/NCD care in clinics. NCD = non-communicable disease.

### Drug costs

We report costs associated with 1) drug costs, 2) laboratory costs and 3) staffing and human resources. Table 2 illustrates that the primary drug expense for PLHIV is attributed to antiretrovirals. Antiretroviral drugs constitute 90% of total drug costs for Clinic A and 75% for Clinic B, assuming all patients are stable and without complications. The drugs included are primarily first-line drugs per national treatment guidelines per condition listed.^17–19^ Additionally, the prices are negotiated for public healthcare facilities and may not reflect market rates.

**TABLE 2.** Drug cost categories associated with integrated HIV/NCD care in clinics per month.

Drug description	Quantity	Medical condition targeted	Clinic A (USD)	Clinic B (USD)
Abacavir, lamivudine, tenofovir	50 mg/300 mg/300 mg; 28 tablets	HIV	6 518,84	3 348,62
Dolutegravir	50 mg; 30 tablets	HIV	2 435,46	1 251,05
Hydrochlorothiazide	12.5 mg; 28 tablets	Hypertension	427,80	698,81
Enalapril	10 mg: 30 tablets	Hypertension	87,35	142,33
Amlodipine	5 mg; 28 tablets	Hypertension	69,36	113,01
Metformin	500 mg; 56 tablets	Diabetes	350,51	619,70
Total drug costs, USD			9 889,32	6 173,52

NCD = non-communicable disease; USD = US dollar.

### Laboratory costs

Table 3 shows the annual laboratory test costs associated with integrated care, focusing on standard tests. Viral load testing is notably the most expensive individual category for both health facilities, while histology for cervical cancer screening represents the least costly category. However, when combined, the liver function tests are more expensive than the viral load test. Clinic A has a higher patient volume, so total laboratory costs are higher.

**TABLE 3.** Laboratory cost categories associated with integrated HIV/NCD care in clinics.

Name of test	Description	Clinic A	Clinic B
		(USD)	(USD)
Viral load	HIV: viral load	42 655.22	21 911,26
CD4 count	HIV: immunity	22 826,37	11 725,52
Glutamyl transferase	Liver function	10 648,57	11 222,76
Alanine transaminase	Liver function	10 625,18	11 198,11
Phosphatase alkaline	Liver function	10 122,34	10 668,15
Serum bilirubin	Liver function	10 122,34	10 668,15
Total cholesterol	Risk factor for cardiovascular disease	10 556,71	11 136,49
Albumin	Cell/tissue function	9 409,01	9 916,35
Total protein	Cell/tissue function	6 099,60	6 428,50
Creatine	Kidney function	7 098,27	7 481,02
Glycated haemoglobine	Diabetes	3 403,13	6 016,68
Histology	Cervical cancer	907,61	302,54
Total laboratory costs, USD		155 099,53	118 650,88

NCD = non-communicable disease; USD = US dollar.

### Estimated annual costs for integrated HIV/NCD care

Table 4 presents the total estimated costs per category per annum. Personnel costs rank highest for both facilities, followed by laboratory tests and drug costs, respectively. The annual estimated cost per patient is USD261.60 for Clinic A and USD226.30 for Clinic B. This suggests that, despite differences in patient load and staffing, the costs of the two health facilities are comparable.

**TABLE 4.** The estimated annual costs of integrating HIV and NCD care in the two study clinics.

Item	Clinic A	Clinic B
Personnel costs	707 235.6 (60.1%)	432 895.4 (57.7%)
Costs of laboratory tests	155 099.53 (13.2%)	118 650.88 (15.8%)
Drug costs	118 671.84 (10.1%)	74 082.24 (9.9%)
Overhead costs	19 6201.4 (16.6%)	12 5125.7 (16.7%)
Estimated total costs of integrating HIV and NCD care	1 177 208.37 (100%)	750 754.22 (100%)
Total costs per patient per year, USD	261.60	226.30

NCD = non-communicable disease; USD = US dollar.

## DISCUSSION

We conducted a comparative case study of two PHC facilities in South Africa to ascertain the cost of integrated care for HIV patients with comorbidities. Specifically, we focused on examining baseline costs without complications.

Analysing two publicly funded PHC facilities, we found that despite differences in on-site resources, both facilities had similar annual estimated costs per patient (USD261.60 and USD226.30) from the provider’s perspective. Our investigation revealed a lack of comparable studies conducted in economic settings similar to ours that we could use to compare our findings. However, a study on cervical cancer screening among HIV-positive women in Johannesburg showed screening costs ranging from USD3.67 to USD54.34, depending on the screening method used.^20^

Notably, a cohort study conducted in Tanzania and Uganda^21^ demonstrated that the costs of integrated care (HIV/NCDs) were lower than managing the chronic conditions in a patient separately. Additionally, prior research has also indicated that incorporating NCD care into HIV programmes can increase costs by 1 to 30%.^6^ Hence, there is a crucial need for context-specific costing of integrated HIV/NCD services to guide healthcare systems.

Our study employed the ABC method, allowing us to factor in personnel, laboratory diagnostics, drug, and overhead costs to estimate the cost of integrated HIV/NCD care based on resource utilisation.

Moreover, negotiated drug pricing presents a significant opportunity for the Southern African region. Bulk purchases can provide leverage for African countries, particularly within the public healthcare system, enabling more favourable negotiations and potentially reducing healthcare costs while considering drug manufacturing solutions within the region.^22^

### Limitations of the study

The study revealed that integrated care costs were USD261.60 and USD226.30 per patient annually at two PHC facilities in South Africa. However, the Department of Health designated these facilities as ‘ideal’ clinics, potentially making them atypical compared to regular PHC facilities nationwide. Additionally, the study only accounted for provider costs, excluding capital and patient-related expenses. Furthermore, we did not capture costs related to treatment complications. Due to limited resources, we could not expand our study to include additional sites, potentially limiting the representativeness of our findings across the entire country.

Moreover, normative costs for integrated HIV/NCD care in countries similar to South Africa, such as Brazil, Russia, India, China and South Africa (BRICS) member states, were unavailable, limiting the study’s ability to provide comparative insights. Data limitations also prevented comparing costs before and after implementing integrated health services at the studied PHC facilities.

### Directions for future research

Future studies should delve into more granular costing of the integrated HIV/NCD care systems, possibly implementing the ABC method within these programs. This approach could offer healthcare managers and researchers valuable costing data, aiding in informed decision-making and resource allocation. The persistent challenge lies in obtaining accurate cost estimates, which is crucial for our constrained budgets and prioritising activities at PHC facilities. Future studies should also consider longitudinal costing studies that can provide data over time, unlike cross-sectional studies that provide data only for specific points in time. Future studies should include multiple healthcare facilities nationwide to enhance the generalizability of cost data, thus increasing the relevance of the National Strategic Plan 2023-2028.

Additionally, there is a pressing need for multi-country costing studies of integrated HIV/NCD care in the African region to generate more generalisable data for efficient resource allocation in under-resourced healthcare systems. Health departments should establish cost databases to facilitate, plan, and budget for various services, with methods like ABC being a potential tool. Increased research is warranted to investigate the costs of integrated HIV/NCD services at the primary healthcare level, further informing healthcare policies and practices.

## Footnotes

* The South African National Department of Health (NDOH) established a programme to create ‘ideal clinics’ which are defined as a ‘clinic with good infrastructure, adequate staff, adequate medicine and supplies, good administrative processes, and sufficient adequate bulk supplies.’ These clinics must excel in ten monitored components: administration, integrated clinical services management, medicines, supplies and laboratory services, human resources for health, support services, infrastructure, health information management, communication, district health system support and implementing partners and stakeholders. To attain ideal clinic status, specific minimum percentages according to the weighted categories must be achieved as outlined in the NDOH ideal clinic framework. Within the context of the current paper, reference to ideal clinics is synonymous with the provision of integrated care.
